# Effect of Immune-Enhancing Enteral Nutrition Enriched with or without Beta-Glucan on Immunomodulation in Critically Ill Patients

**DOI:** 10.3390/nu8060336

**Published:** 2016-06-02

**Authors:** Jae Gil Lee, Young Sam Kim, Young Ju Lee, Hyeon Yeong Ahn, Minjoo Kim, Minkyung Kim, Min Jung Cho, Younsoo Cho, Jong Ho Lee

**Affiliations:** 1 Department of Surgery, Yonsei University College of Medicine, Seoul 03722, Korea; jakii@yuhs.ac; 2 Department of Internal Medicine, Yonsei University College of Medicine, Seoul 03722, Korea; ysamkim@yuhs.ac; 3 National Leading Research Laboratory of Clinical Nutrigenetics/Nutrigenomics, Department of Food and Nutrition, College of Human Ecology, Yonsei University, Seoul 03722, Korea; juny9558@naver.com (Y.J.L.); mkkim0106@yonsei.ac.kr (M.K.); wm5156@hanmail.net (M.J.C.); 4 Department of Food and Nutrition, Brain Korea 21 PLUS Project, College of Human Ecology, Yonsei University, Seoul 03722, Korea; 5 Research Center for Silver Science, Institute of Symbiotic Life-TECH, Yonsei University, Seoul 03722, Korea; vitaminahy@naver.com (H.Y.A.); minjookim@yonsei.ac.kr (M.K.); 6 Department of Nutrition, Yonsei University Health System, Seoul 03722, Korea; yscho@yuhs.ac

**Keywords:** beta-glucan, enteral nutrition, ICU, NK cell, immune system, inflammation

## Abstract

We investigated whether high-protein enteral nutrition with immune-modulating nutrients (IMHP) enriched with β-glucan stimulates immune function in critically ill patients. In a randomized double-blind placebo-controlled study, 30 patients consumed one of three types of enteral nutrition: a control or IMHP with and without β-glucan. The IMHP with β-glucan group showed increases in natural killer (NK) cell activities relative to the baseline, and greater increases were observed in NK cell activities relative to the control group after adjusting for age and gender. The IMHP groups with and without β-glucan had greater increases in serum prealbumin and decreases in high-sensitivity C-reactive protein (hs-CRP) than the control group. The control group had a greater decrease in peripheral blood mononuclear cell (PBMC) interleukin (IL)-12 production than the IMHP with and without β-glucan groups. In all patients, the change (Δ) in hs-CRP was correlated with Δ prealbumin and Δ PBMC IL-12, which were correlated with ΔNK cell activity and Δ prealbumin. This study showed beneficial effects of a combination treatment of β-glucan and IMHP on NK cell activity. Additionally, strong correlations among changes in NK cell activity, PBMC IL-12, and hs-CRP suggested that β-glucan could be an attractive candidate for stimulating protective immunity without enhanced inflammation (ClinicalTrials.gov: NCT02569203).

## 1. Introduction

Critically ill patients are at risk of nutritional deficiency; thus, supportive nutrition is required for most intensive care unit (ICU) patients, with enteral nutrition preferred over parenteral nutrition [1,2]. Enteral nutrition with immune-modulating nutrients, such as ω-3 fatty acids, selenium, and antioxidants, may modulate pathophysiological processes in critical illness, such as inflammatory and oxidative stress responses and impaired immune function [3,4]. The immunomodulatory effect of the *Lentinula edodes* (shiitake) mushroom has been conducted in previous studies and there are various biological active compounds in mushrooms. β-glucan, which is derived from mushrooms, is known as one of the biological active compounds in mushrooms [5,6,7,8]. Recently, β-glucan polysaccharides have been reported to stimulate the immune system, modulating humoral and cellular immunity and thereby having beneficial effects in fighting infections. Previous clinical studies conducted the immunomodulatory effects of β-glucan in patients with cancer, allergies, or respiratory tract infection [9,10,11]. Richter *et al.* reported that short-term oral application of β-glucan significantly stimulated mucosal immunity of children with chronic respiratory problems in a series of clinical trials [12,13]. β-glucan is thought to mediate its stimulatory effects through the activation of various immune system components, including macrophages, neutrophils, natural killer (NK) cells, and lymphocytes [14,15,16,17].

Although previous data clearly provide support for an immunomodulatory effect of β-glucan, there are few clinical studies on the immunomodulatory effect of β-glucan in critically ill patients. More clinical data are therefore clearly needed on the efficacy of orally supplemented β-glucan as an immune modulator in critically ill patients. The objective of this study was to determine whether high-protein (24% of total calories from protein) enteral nutrition of immune-modulating nutrients (e.g., ω-3 fatty acids, selenium, and antioxidants) (IMHP) enriched with β-glucan stimulates immune function compared with standard enteral nutrition (control: 20% of total calories from protein) or IMHP without β-glucan in critically ill patients.

## 2. Materials and Methods

### 2.1. Participants

From April 2014 to September 2015, 30 critically ill patients were enrolled in this study after admission to the ICU at Yonsei University Severance Hospital. The ICU patients were composed of 18 patients with pulmonary disease and 12 patients with trauma. Disease severity was evaluated by the Acute Physiology and Chronic Health Evaluation (APACHE) II score [18]. All patients were treated according to the appropriate guidelines [19,20]. Informed consent was provided by a close family member. This investigation was approved by the Institutional Review Board at Yonsei University Severance Hospital, Seoul, Korea (Approval number: 4-2013-0902). All comorbidities and histories of the study participants were recorded (ClinicalTrials.gov: NCT02569203) [21].

### 2.2. Randomization and Intervention

Using computer-generated randomization lists, 30 critically ill patients were randomized to receive one of three types of enteral nutrition: standard enteral nutrition (control), high-protein enteral nutrition with immune-modulating nutrients (IMHP) enriched with β-glucan, or IMHP without β-glucan. The ready-to-use control and IMHP with and without β-glucan products had identical packaging with no differences in appearance, texture, or smell. Investigators and clinicians were blinded to the treatment groups. Patients assigned to the control group received a standard formula tube feed (protein:fat:carbohydrate from total calories = 20%:30%:50%; Dr. Chung’s Food Co., LTD, Cheongju, Korea). Those assigned to the IMHP group received ω-3 fatty acid (3.3 g/L)- and antioxidant (110 μg/L selenium)-enriched high protein tube feed (protein:fat:carbohydrate from total calories = 24%:30%:46%; Dr. Chung’s Food Co., LTD, Korea). Those assigned to the IMHP group with β-glucan received β-glucan-enriched IMPH tube feed (experimental product; Dr. Chung’s Food Co., LTD, Korea).

β-glucan derived from mushrooms (*Lentinus edodes*) and the content of β-glucan in mushroom extract was 13%. The β-glucan used in this study was not concentrated; instead, the whole mushroom extract was included in enteral nutrition product for IMHP with β-glucan group. IMHP with β-glucan group was designed to contain 50 mg of β-glucan per 200 kcal (0.25 mg/kcal) from the mushroom extract, and the rest was contents to the standard formula used in the control group, but fortified with ω-3 fatty acid, antioxidants, and enriched high protein. The enteral nutrition product for IMHP group was designed to contain exactly the same contents as those for the IMHP group with β-glucan except for the β-glucan content. The composition of the enteral nutrition product was indicated in Table 1.

Enteral nutrition was initiated within 24 h of ICU admission. Enteral feeding was delivered at a constant rate to achieve a minimum of 50% basal energy expenditure (BEE; determined using the Harris–Benedict equation) × 1.2 within the first 12 h [22]. If well tolerated, enteral nutrition was advanced to achieve a BEE × 1.2 within 48 h. Complementary feeding with enteral or parenteral nutrition was allowed for an initial 48 h. From the third day, the patient received a minimum of 75% of BEE × 1.2. The enteral diet was delivered continuously for seven days during the ICU stay at a rate not exceeding BEE × 1.2. The daily enteral intake was recorded to obtain the total volume and calories delivered to the patients. The data on daily total calorie intake were abstracted from the medical records. Blood samples were collected at the baseline and after seven days.

### 2.3. Anthropometric Parameters and Blood Collection

Body mass index (BMI) was measured with an Inbody S10 bedside-type body composition analyzer (Inbody, Cheonan, Republic of Korea) in a supine state in the morning. Venous blood specimens were collected in EDTA-treated and plain tubes and centrifuged to obtain plasma and serum. The collected blood samples were stored at −70 °C until analysis.

### 2.4. Serum Lipid Profiles and Glucose

Serum triglyceride and serum total cholesterol concentrations were analyzed by enzymatic assays using a Hitachi 7600 autoanalyzer (Hitachi, Tokyo, Japan). Serum high-density lipoprotein (HDL)-cholesterol concentrations were determined by selective inhibition using a Hitachi 7600 autoanalyzer. Low-density lipoprotein (LDL)-cholesterol concentrations were calculated indirectly using the Friedwald formula; *i.e.*, LDL-cholesterol = total cholesterol − [HDL-cholesterol + (triglyceride/5)] for subjects with serum triglyceride concentrations <400 mg/dL. Serum glucose concentrations were measured according to the hexokinase method on a Hitachi 7600 autoanalyzer.

### 2.5. Serum Nutritional Status

Serum albumin concentrations were analyzed through the BCG method using an ALB kit (Siemens, Tarrytown, NY, USA) with an ADVIA 2400 autoanalyzer (Siemens, Tarrytown, NY, USA). Serum prealbumin concentrations were determined by an immunoturbidimetric assay using a COBAS INTEGRA autoanalyzer (Roche-BM, Rotkreuz, Switzerland).

### 2.6. Serum Liver and Renal Function

Serum glutamic oxaloacetic transaminase (GOT) and serum glutamic pyruvate transaminase (GPT) were analyzed through the IFCC UV method with a Hitachi 7600 autoanalyzer. Serum gamma-glutamyl transpeptidase (γ-GTP) was measured according to a modified Szanz method on a Hitachi 7600 autoanalyzer. Blood urea nitrogen (BUN) was determined by a kinetic UV assay for urea/urea nitrogen using a Hitachi 7600 autoanalyzer. Creatinine was analyzed through the creatinine Jaffe method on a Hitachi 7600 autoanalyzer.

### 2.7. Leukocyte Count and Serum High-Sensitivity C-Reactive Protein

Leukocyte count was determined using the HORIBA ABX diagnostic analyzer (HORIBA ABX SAS, ParcEuromedicine, Montpellier, France). Serum high-sensitivity C-reactive protein (hs-CRP) levels were measured with a kit from the N-Assay LA CRP-S D-TYPE (Nittobo, Tokyo, Japan) with a Hitachi 7600 autoanalyzer.

### 2.8. Peripheral Blood Mononuclear Cells

To analyze the cytokine assay in peripheral blood mononuclear cells (PBMCs) supernatants, we isolated PBMCs from whole blood samples. Whole blood samples were mixed with the same volume of RPMI 1640 (Gibco, Thermo Fisher Scientific, Waltham, MA, USA) and gently overlaid on the histopaque (Sigma-Aldrich, Irvine, UK) and then centrifuged (20 min, 1800 rpm, 15 °C). After separation, a thin layer of buffer coat was isolated and washed twice with RPMI 1640. The pellet was resuspended in RPMI 1640 supplemented with penicillin streptomycin (Gibco, Thermo Fisher Scientific, Waltham, MA, USA). The isolated PBMCs were cultured in RPMI 1640 supplemented with 10% fetal bovine serum (Gibco, Thermo Fisher Scientific, Waltham, MA, USA), seeded into 12-well plates (1.0 × 10^6^ cells/mL), and incubated at 37 °C under 5% CO_2_ for no more than 46 h ± 30 min. After incubation, the supernatants were collected and stored at −80 °C.

### 2.9. Cytokine Assay in Serum and PBMC Supernatants

Interferon (IFN)-γ was measured with a kit from an IFN gamma High-Sensitivity Human ELISA Kit (Abcam plc-Cambridge Science Park, Cambridge, UK) according to the manufacturer’s instructions. Interleukin (IL)-12 levels were analyzed by a High-Sensitivity Human IL-12 (P70) ELISA kit (Genway Biotech Inc., San Diego, CA, USA) using a Victor ×5 2030 multilabel plate reader (PerkinElmer, Hopkinton, MA, USA) at 450 nm. IL-6, IL-1β, and tumor necrosis factor (TNF)-α levels in serum and PBMC supernatants were measured using the Bio-Plex™Reagent Kit (Bio-Rad Laboratories, Hercules, CA, USA).

### 2.10. NK Cell Activity

Isolated PBMCs from the whole blood samples were incubated with K562 cells to analyze the cytotoxic activity of NK cells. A whole blood sample was mixed with the same volume of RPMI medium 1640 (Gibco, Thermo Fisher Scientific, Waltham, MA), then gently overlaid on Histopaque^®^1077 (Sigma-Aldrich, Irvine, UK) and centrifuged for 20 min at 1800 rpm at 15 °C. After separation, a buffer coat layer was isolated, washed once with RPMI 1640 medium, and then resuspended in 1 mL of 10% fetal bovine serum. The isolated PBMCs (effector cell, E) were seeded into 96-well plates at ratios of 5:1 and 2.5:1 with the K562 cells (2 × 10^4^ cells/well) (target cell, T) and then incubated at 37 °C under 5% CO_2_ for more than 4 h. The cytolytic activities of NK cells were analyzed via the CytoTox 96^®^ Non-Radioactive Cytotoxicity Assay Kit (Promega Co., Fitchburg, WI, USA) according to the manufacturer’s instructions. The color reactions were read at 490 nm using a Victor ×5 2030 multilabel plate reader (PerkinElmer, Hopkinton, MA, USA), and the results were calculated by the following formula:

$$\%\text{ Cytotoxicity }=\frac{\text{Experimental-Effector Spontaneous-Target Spontaneous}}{\text{Target Maximum-Target Spontaneous}}\times 100$$

### 2.11. Statistical Analysis

Statistical analysis was performed using SPSS version 21.0 (IBM/SPSS Corp., Chicago, IL, USA). The logarithmic transformation was performed on skewed variables. We compared the parameters at the baseline and follow-up, and net change (difference from the baseline) among the control, IMHP enriched with β-glucan. And IMHP groups by using the *Kruskal–Wallis* test and the *Mann–Whitney* U-test with *Bonferroni* correction. The *Wilcoxon* test was evaluated to compare the effects of the intervention within each group. A general linear model test was applied to adjust for potential confounding factors. The Spearman correlation coefficient was used to examine relationships between variables. A heat map was generated to visualize correlations among variables. The results were expressed as the mean ± standard error (SE). A *p*-value < 0.05 was considered statistically significant.

## 3. Results

### 3.1. Clinical Characteristics

This study enrolled 30 patients. Eight patients (three control, two IMHP with β-glucan, and three IMHP) dropped out of the study. The major reasons for exclusion were the inability to meet the caloric goal, withdrawn consent for the clinical trial, and death by multiple organ failure. Table 2 shows the general and biochemical characteristics of the control and IMHP with and without β-glucan groups. No statistically significant differences among the three groups were observed at the baseline with regard to age, gender distribution, APACHE II score, and total calorie intake (day 1, day 3, and day 7). Estimated average daily intake of β-glucan was 232.8 mg in IMHP with β-glucan group. After adjustment for age and gender distribution, no significant differences among the three groups were observed at the baseline and at the seven-day follow-up with regard to BMI, serum glucose, triglycerides, total cholesterol, HDL, LDL, leukocyte counts, and serum albumin. After seven days of treatment, patients in the IMHP group showed significant increases in serum concentrations of total cholesterol, LDL cholesterol, and albumin and patients in the control group showed a significant elevation in leukocyte counts (Table 2).

### 3.2. Effects on NK Cell Activity and Serum Prealbumin Following Seven Days of Tube Feeding of the Control and the IMHP Groups with and without β-Glucan

NK cell activities (%) were measured based on E:T ratios of 5:1 or 2.5:1. As shown in Table 3, no significant differences were found in the NK cell activities in both conditions measured at the baseline among the three groups. NK cell activities at the 5:1 or 2.5:1 E:T ratios were significantly increased in the IMHP with β-glucan group at seven days compared to the baseline. When we compared the changes among the three groups, the IMHP with β-glucan group had greater increases in NK cell activity at a ratio of E:T = 5:1 than the control group before (*p* = 0.019) and after (*p* = 0.037) adjusting for age and gender. Additionally, the IMHP with β-glucan group had greater increases in NK cell activity at ratio of E:T = 2.5:1 (*p* = 0.034) than the control group; however, only an increasing tendency remained after adjusting for age and gender distribution (*p* = 0.055).

No significant differences were found in serum prealbumin concentrations at the baseline among the three groups (Table 3). Serum prealbumin concentrations were significantly increased in the IMHP with β-glucan group and IMHP group at seven days compared to the baseline. When we compared the changes among the three groups, the IMHP with β-glucan group and IMHP group had greater increases in serum prealbumin concentrations than the control group before (*p* = 0.002) and after (*p* = 0.001) adjusting for age and gender. The IMHP group had the greatest increase in serum prealbumin concentrations among the three groups. At seven days, serum prealbumin concentrations were higher in the IMHP group than those in the IMHP with β-glucan group and the control group (*p* = 0.017).

### 3.3. Effects on Serum CRP and Cytokines and PBMC Cytokine Production Following Seven Days of Tube Feeding in the Control and the IMHP Groups with and without β-Glucan

As shown in Table 3, no significant differences were found in serum hs-CRP and cytokines and PBMC cytokine production at the baseline among the three groups. Serum CRP concentrations were significantly decreased in the IMHP with β-glucan group and the IMHP group at seven days compared to the baseline. When we compared the changes among the three groups, the IMHP with β-glucan group and the IMHP group had greater decreases in serum CRP concentrations than the control group before (*p* = 0.002) and after (*p* = 0.006) adjusting for age and gender. The IMHP group had the greatest decrease in serum CRP concentrations among the three groups. At seven days, serum CRP concentrations were lower in the IMHP group than those in the IMHP with β-glucan group and control group (*p* = 0.006).

PBMC IL-12 production was significantly decreased in the control group at seven days compared to the baseline. When we compared the changes in PBMC IL-12 among the three groups, the control group had a greater decrease in IL-12 release from PBMC than the IMHP with β-glucan group and the IMHP group before (*p* = 0.004) and after adjusting for age and gender (*p* = 0.003). At seven days, the PBMC IL-6 level was significantly higher in the control group than in the IMHP group (*p* = 0.046) (Table 3).

### 3.4. Relationships among Changes in BMI, Serum Albumin, Prealbumin, Cytokines, PBMC Cytokine Production, and NK Cell Activity

**Figure 1:**
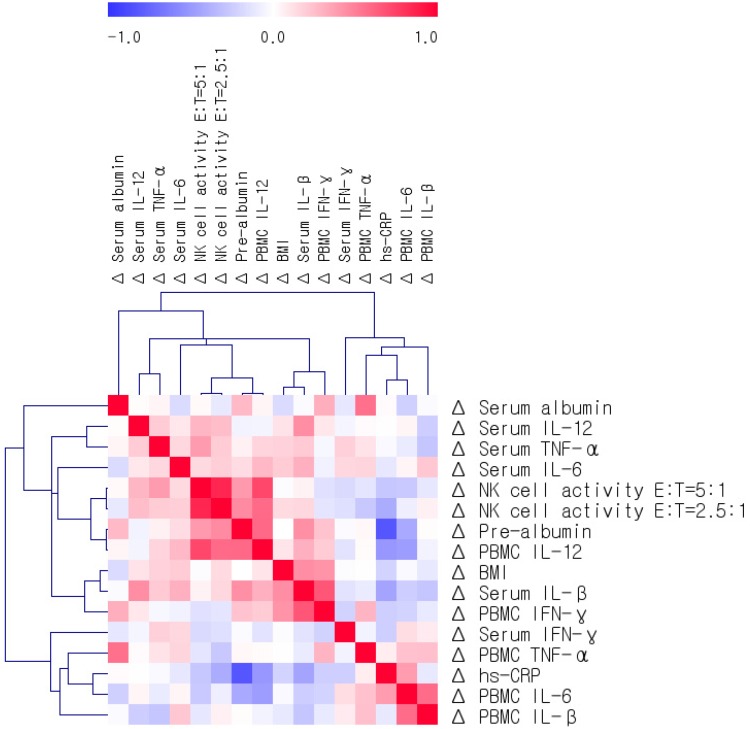
Matrix of correlations among changes in BMI, serum albumin, pre-albumin, cytokines, PBMC cytokine production, and NK cell activity. Correlations were obtained by deriving Spearman’s correlation coefficient. *Red* is a positive correlation and *blue* is a negative correlation.

**Figure 2:**
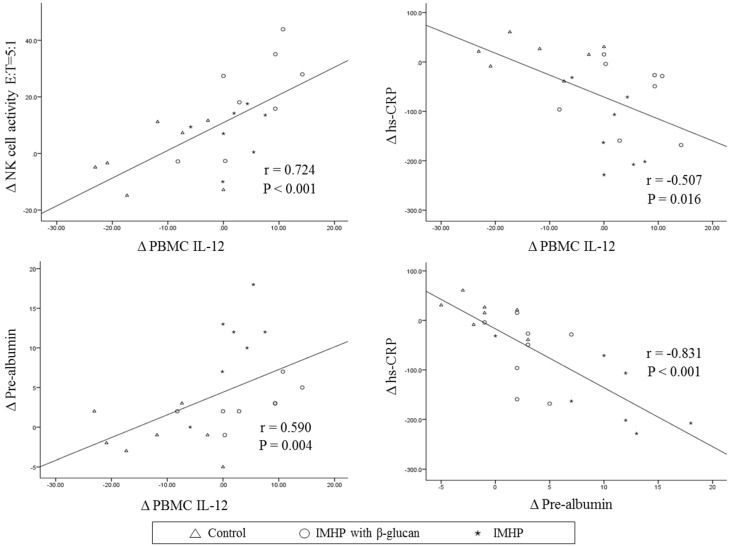
Correlations among changes in NK cell activity E:T = 5:1, PBMC IL-12, serum hs-CRP, and serum pre-albumin in all subjects. Tested by Spearman’s correlation analysis, r: correlation coefficients.

Correlations among the changed levels (Δ) of BMI, serum albumin, prealbumin, cytokines, PBMC cytokine production, and NK cell activity were determined after adjusting for age and gender (Figure 1). In 22 patients, Δ BMI positively correlated with Δ serum IL-1β, which was positively correlated with Δ serum IL-12 and Δ PBMC IFN-γ. The net Δ of hs-CRP was strongly and negatively correlated with Δ serum prealbumin (*r* = −0.831, *p* < 0.001) and Δ PBMC IL-12 (*r* = −0.507, *p* = 0.016) (Figure 2). The net Δ of serum albumin was positively correlated with Δ PBMC TNF-α and Δ serum prealbumin was positively correlated with ΔNK cell activity (E:T = 5:1) and Δ PBMC IL-12 (*r* = 0.590, *p* = 0.004) (Figure 2). ΔNK cell activity (E:T = 5:1) was strongly and positively correlated with Δ NK cell activity (E:T = 2.5:1) (*r* = 0.831, *p* < 0.001) and ΔNK cell activity at both conditions was positively correlated with Δ serum prealbumin and Δ PBMC IL-12. Δ Serum TNF-α was positively correlated with Δ NK cell activity (E:T = 5:1). Δ PBMC IL-6 was negatively correlated with Δ PBMC IL-12 and positively correlated with Δ PBMC IL-1β (Figure 2).

## 4. Discussion

This randomized double-blind placebo-controlled study found beneficial effects following enteral nutrition with a combination of β-glucan (250 mg/L) and IMHP [high-protein (24% of total calories from protein). These immune-modulating nutrients (e.g., ω-3 fatty acids and antioxidants) had beneficial effects on NK cell activity, a marker of immune competence [23,24,25], in critically ill patients known to exhibit hyporesponsiveness of NK cell activity [26]. This result is in agreement with those of a previous study in which β-glucan enhanced NK cell activation in mice [27,28]. Generally, the evidence in immune modulation by β-glucan is strongly supported by numerous studies [16,17]. However, there are few clinical studies to demonstrate the immunomodulatory effects of orally supplemented β-glucan in critically ill patients. In this clinical trial, the IMHP with the β-glucan group showed significant increases in NK cell activities at 5:1 or 2.5:1 E:T ratios from the baseline, and a significantly greater increase was seen in those at the 5:1 E:T ratio than in the control group. Additionally, an increase in NK cell activity at a ratio of E:T = 2.5:1 showed a greater tendency in the IMHP with β-glucan group than the control group after adjusting for age and gender. These results could suggest a synergistic effect of β-glucan on enhanced NK cell activity following seven days of feeding of IMHP enriched with β-glucan in comparison with IMHP without β-glucan.

The recent report that NK cells have beneficial anti-infection and anti-inflammatory properties [29] supports the idea that therapeutic immune intervention in critically ill patients could stimulate the function of NK cells [30]. The immunostimulating effect of β-glucan is associated with the activation of NK cells, macrophages, and T-helper (Th) cells [5]. Activated macrophages/monocytes release IL-12, which may exert a protective effect in critically ill patients through the IL-12-induced increase in cellular immunity and phagocytic functions [31]. In the control group of this study, IL-12 release from PBMC significantly decreased from the baseline, but the IMHP with β-glucan group showed a slight but not significant increase in PBMC IL-12 production and serum IL-12 concentration. Additionally, changes in NK cell activities were positively correlated with changes in PBMC IL-12 levels.

IL-12 positively regulates IFN-γ secretion by NK cells, which is a major source of IFN-γ, a potent immune stimulatory cytokine [25]. In this study, however, there were no significant changes in the serum and PBMC levels of IFN-γ, TNF-α, IL-6, and IL-1β in the IMHP groups with and without β-glucan. Additionally, changes in PBMC IL-12 were negatively correlated with changes in serum hs-CRP. Furthermore, the IMHP groups with and without β-glucan showed a greater reduction in hs-CRP than the control group, which showed the highest serum hs-CRP and PBMC IL-6 production among the three groups at seven days and increased leukocyte counts from the baseline. IMHP groups with and without β-glucan contained 3.3 g/L ω-3 fatty acids, which are known to suppress leukocyte numbers, cytokine production, and lymphocyte proliferation [32]. Therefore, the lack of changes in serum and PBMC IFN-γ, TNF-α, IL-6, and IL-1β, even with increased NK-cell activities in the IMHP with β-glucan group, could be related to the intake of ω-3 fatty acid.

Changes in serum levels of hs-CRP were negatively correlated with changes in serum prealbumin. As expected, serum prealbumin indicated higher protein intake in the IMHP groups with and without β-glucan (24% of total calorie intake from protein) than in the control group (20% of total calorie intake from protein). Additionally, changes in serum prealbumin were positively correlated with changes in PBMC IL-12, one of the Th1 cytokines, to increase cellular immunity [31]. These results partly support previous findings that nutrition therapy in critically ill patients plays an important role in assisting recovery and improving outcomes [32,33,34,35,36]. In spite of the small sample size of 22 critically ill patients, this randomized double-blind placebo-controlled study clearly showed beneficial effects following a combination of β-glucan (250 mg/L) and IMHP [high-protein (24% of total calorie from protein) enteral nutrition of immune-modulating nutrients (e.g., ω-3 fatty acids and antioxidants)] on NK cell activity, a marker of immune competence, without significant changes in serum and PBMC levels of IFN-γ, TNF-α, IL-6, and IL-1β. Additionally, a strong positive correlation between changes in NK cell activity and changes in PBMC IL-12, which were negatively correlated with changes in hs-CRP, suggests that β-glucan could be an attractive candidate to be added to IMHP for the stimulation of protective immunity without enhancing inflammation. Further investigations are required to evaluate the effect of β-glucan on mortality and complication rates in larger long-term trials.

## 5. Conclusions

Immune-enhancing enteral nutrition enriched with β-glucan showed significant increases in NK cell activities from the baseline and a significantly greater increase than the control group. IMHP with and without β-glucan had greater increases in serum albumin and decreases in hs-CRP than the control group. The control group had a greater decrease in PBMC IL-12 production than the IMHP groups with and without β-glucan. This study showed the beneficial effects of a combination treatment of β-glucan and IMHP on NK cell activity, and suggested that β-glucan could be an attractive candidate to add to IMHP for stimulation of protective immunity without enhanced inflammation.

## Figures and Tables

**Table 1:** Composition of the enteral nutrition product.

Nutrient	Control	IMHP with β-Glucan	IMHP
Calories (kcal)	200	200	200
Protein (g)	10.0	12.0	12.0
Total fat (g)	6.7	6.7	6.7
Total carbohydrate (g)	28.5	23.0	23.0
Vitamin A (μgRE)	150.00	150.00	150.00
VitaminB1 (mg)	0.24	0.24	0.24
VitaminB2 (mg)	0.30	0.30	0.30
VitaminB6 (mg)	0.30	0.30	0.30
VitaminB12 (μg)	0.48	0.48	0.48
Vitamin C (mg)	20.00	40.00	40.00
VitaminD3 (μg)	1.00	1.00	1.00
Vitamin E (mgα-TE)	2.00	4.80	4.80
VitaminK1 (μg)	9.75	15.00	15.00
Folic acid (μg)	80.00	80.00	80.00
Niacin (mg)	3.20	3.20	3.20
Biotin (μg)	6.00	6.00	6.00
Pantothenic acid (mg)	1.00	1.00	1.00
Calcium (mg)	140.00	150.00	150.00
Phosphorus (mg)	140.00	140.00	140.00
Magnesium (mg)	58.00	44.20	44.20
Zinc (mg)	2.00	4.00	4.00
Iron (mg)	2.00	2.00	2.00
Sodium (mg)	155.00	141.35	141.35
Chloride (mg)	170.00	121.20	121.20
Potassium (mg)	260.00	240.39	240.39
Manganese (mg)	0.46	1.60	1.60
Iodine (μg)	19.50	30.00	30.00
Copper (mg)	0.10	0.32	0.32
Selenium (μg)	0.00	22.00	22.00
Chromium (μg)	0.00	5.00	5.00
Molybdenum (μg)	0.00	2.50	2.50
Taurine (mg)	22.00	22.00	22.00
L-Carnitine (mg)	22.00	22.00	22.00
Choline (mg)	73.00	73.00	73.00
β-glucan (mg)	0.00	50.00	0.00

**Table 2:** Clinical characteristics in the control and in IMHP groups with and without β-glucan.

Variables	Total (*n* = 22)						*p^a^*	*p^b^*
	Control (*n* = 7)		IMHP with β-Glucan (*n* = 8)		IMHP (*n* = 7)			
	Baseline	Follow-up	Baseline	Follow-up	Baseline	Follow-up		
Age (year)	53.7 ± 6.18		64.1 ± 6.03		72.6 ± 3.23		0.141	
Male/Female n, (%)	4 (57.1)/3 (42.9)		5 (62.5)/3 (37.5)		6 (85.7)/1 (14.3)		0.488	
BMI (kg/m^2^)	24.1 ± 1.31	23.4 ± 1.38	22.1 ± 1.20	21.9 ± 1.37	20.8 ± 1.50	20.2 ± 1.39	0.213	0.114
APACHE II score	16.7 ± 3.07		16.9 ± 3.39		18.5 ± 2.69		0.862	
TCI (kcal/d), mean								
Day 1	736.3 ± 176.0		738.1 ± 211.5		790.3 ± 129.1		0.688	
Day 3	1253.5 ± 214.3		1191.0 ± 69.8		1059.3 ± 151.1		0.546	
Day 7	1507.7 ± 157.2		1065.9 ± 153.3		1102.0 ± 156.7		0.170	
Glucose (mg/dL)*^∮^*	136.1 ± 8.40	143.1 ± 5.86	179.0 ± 29.3	173.8 ± 29.1	306.9 ± 117.4	154.6 ± 17.4	0.052	0.959
Triglyceride (mg/dL)*^∮^*	104.0 ± 19.6	79.3 ± 8.83	116.3 ± 16.3	109.0 ± 12.1	79.0 ± 13.6	84.4 ± 11.2	0.335	0.145
Total-cholesterol (mg/dL)*^∮^*	105.7 ± 10.9	118.4 ± 13.2	108.0 ± 13.2	132.0 ± 8.85	112.0 ± 13.9	145.0 ± 7.75 *	0.998	0.293
HDL-cholesterol (mg/dL)*^∮^*	27.9 ± 6.34	31.6 ± 4.08	25.9 ± 4.67	24.9 ± 3.54	34.6 ± 4.19	38.6 ± 3.88	0.645	0.057
LDL-cholesterol (mg/dL)*^∮^*	57.1 ± 7.76	71.0 ± 12.7	58.9 ± 11.7	85.3 ± 7.69	61.6 ± 12.6	89.4 ± 6.71 *	0.924	0.526
Leukocyte counts (Χ10^3^/μL)*^∮^*	6.99 ± 0.48	12.2 ± 2.26 *	12.3 ± 2.21	12.7 ± 2.24	12.9 ± 1.83	10.8 ± 1.28	0.089	0.839
Serum albumin (mg/dL)*^∮^*	2.71 ± 0.20	2.94 ± 0.21	2.83 ± 0.10	2.94 ± 0.18	2.69 ± 0.23	2.93 ± 0.19 *	0.722	0.979

Mean ± SE. *^∮^* tested by logarithmic transformation, *p^a^*-values derived from *Kruskal–Wallis* test at the baseline. *p^b^*-values derived from *Kruskal–Wallis* test in follow-up. * p < 0.05 derived from *Willcoxon* test. IMHP: high-protein enteral nutrition with immune-modulating nutrients. BMI: body mass index. TCI: total calorie intake. HDL: high-density lipoprotein. LDL: low-density lipoprotein.

**Table 3:** NK cell activity, prealbumin, serum, and PBMC cytokines in the control and the IMHP groups with and without β-glucan.

Variables	Total (*n* = 22)												*p^a^*	*p^b^*	*p^c^*	*p^d^*
	Control (*n* = 7)				IMHP with β-Glucan (*n* = 8)				IMHP (*n* = 7)							
	Baseline		Follow-up		Baseline		Follow-up		Baseline		Follow-up					
NK cell activity 5:1 (%)*^∮^*	13.6 ± 3.69		12.7 ± 5.62		11.0 ± 2.40		31.4 ± 6.36 *		10.7 ± 7.41		18.2 ± 4.63		0.266	0.155		
Change	−0.86 ± 4.17 *^b^*				20.4 ± 5.93 *^a^*				7.47 ± 3.59 *^a,b^*						0.019	0.037
NK cell activity 2.5:1 (%)*^∮^*	14.1 ± 2.14		11.8 ± 3.60		9.88 ± 5.14		29.3 ± 4.76 *		7.56 ± 5.37		18.4 ± 6.11		0.841	0.059		
Change	−2.34 ± 3.08 *^b^*				19.4 ± 6.67 *^a^*				10.8 ± 6.18 *^a,b^*						0.034	0.055
Prealbumin (mg/dL)*^∮^*	11.9 ± 2.15		10.9 ± 1.86 *^b^*		10.4 ± 0.84		13.3 ± 0.80 ^b,^*		9.00 ± 1.21		19.3 ± 1.87 ^a,^*		0.589	0.017		
Change	−1.00 ± 1.05 *^c^*				2.88 ± 0.83 *^b^*				10.3 ± 2.12 *^a^*						0.002	0.001
hs-CRP (mg/dL)*^∮^*	84.7 ± 20.0		99.7 ± 18.9 *^a^*		130.3 ± 33.0		65.7 ± 13.9 ^a,^*		164.1 ± 23.8		19.8 ± 9.87 ^b,^*		0.093	0.006		
Change	15.0 ± 11.9 *^a^*				−64.6 ± 24.5 *^b^*				−144.3 ± 28.5 *^b^*						0.002	0.006
Serum																
IL-12 (pg/mL)	9.54 ± 8.84		7.67 ± 4.58		3.21 ± 3.21		10.6 ± 9.55		3.90 ± 3.90		0.00 ± 0.00		0.696	0.193		
IFN- γ (pg/mL)	0.03 ± 0.03		0.03 ± 0.03		0.24 ± 0.24		0.28 ± 0.28		0.00 ± 0.00		0.00 ± 0.00		0.613	0.613		
TNF-α (pg/mL)*^∮^*	6.23 ± 1.87		3.78 ± 0.46		6.55 ± 1.39		6.06 ± 1.79		7.80 ± 0.81		6.67 ± 1.79		0.889	0.634		
IL-6 (pg/mL)*^∮^*	66.8 ± 23.2		49.5 ± 23.6		39.5 ± 9.94		23.5 ± 6.07		72.3 ± 51.6		13.2 ± 3.02		0.456	0.090		
IL-1β (pg/mL)*^∮^*	7.27 ± 5.65		4.31 ± 3.47		0.82 ± 0.08		0.75 ± 0.07		0.92 ± 0.17		1.05 ± 0.34		0.217	0.321		
Nonstimulated PBMCs																
IL-12 (pg/mL)	24.1 ± 6.06		12.2 ± 5.47 *		15.3 ± 4.73		20.2 ± 4.55		18.8 ± 2.19		20.7 ± 3.16		0.519	0.305		
Change	−11.9 ± 3.38 *^b^*				4.83 ± 2.61 *^a^*				1.89 ± 1.68 *^a^*						0.004	0.003
IFN- γ (pg/mL)	1.00 ± 0.31		0.67 ± 0.19		0.54 ± 0.17		0.75 ± 0.23		0.78 ± 0.18		0.76 ± 0.20		0.520	0.999		
TNF-α (pg/mL)*^∮^*	9.66 ± 4.98		25.4 ± 16.9		3.17 ± 2.06		3.95 ± 0.96		5.71 ± 2.27		3.92 ± 0.99		0.651	0.578		
IL-6 (pg/mL)*^∮^*	26.9 ± 5.19		119.5 ± 70.5 *^a^*		12.1 ± 3.07		21.1 ± 9.65 *^a,b^*		24.2 ± 8.06		12.6 ± 2.25 *^b^*		0.054	0.046		
IL-1β (pg/mL)*^∮^*	4.08 ± 1.49		9.32 ± 5.54		1.93 ± 0.64		2.52 ± 1.15		2.35 ± 0.58		1.87 ± 0.60		0.297	0.312		

Mean ± SE. *^∮^* tested by logarithmic transformation, *p^a^*-values derived from *Kruskal–Wallis* test at the baseline. *p^b^*-values derived from *Kruskal–Wallis* test in follow-up. *p^c^*-values derived from *Kruskal–Wallis* test in Changed value. *p^d^*-values adjusted for age and sex. All alphabetical *p* < 0.05 derived from *Mann–Whitney* U-test with *Bonferroni* correction; no significant differences among the groups marked by the same letter and significant differences among the groups marked with different letters. * p < 0.05 derived from *Willcoxon* test in each group. NK: natural killer. IMHP: high-protein enteral nutrition with immune-modulating nutrients. PBMC: peripheral blood mononuclear cell. hs-CRP: high-sensitivity C-reactive protein. IL: interleukin. IFN: interferon. TNF: tumor necrosis factor.

## Abbreviations

The following abbreviations are used in this manuscript:

BEE	basal energy expenditure
BMI	body mass index
BUN	blood urea nitrogen
E	effector cell
γ-GTP	gamma-glutamyl transpeptidase
GOT	glutamic oxalacetic transaminase
GPT	glutamic pyruvate transaminase
HDL	High-density lipoprotein
hs-CRP	high-sensitivity C-reactive protein
ICU	intensive care unit
IFN	interferon
IL	interleukin
IMHP	high-protein enteral nutrition with immune-modulating nutrients
LDL	Low-density lipoprotein
NK	natural killer
PBMC	peripheral blood mononuclear cell
T	target cell
TNF	tumor necrosis factor
